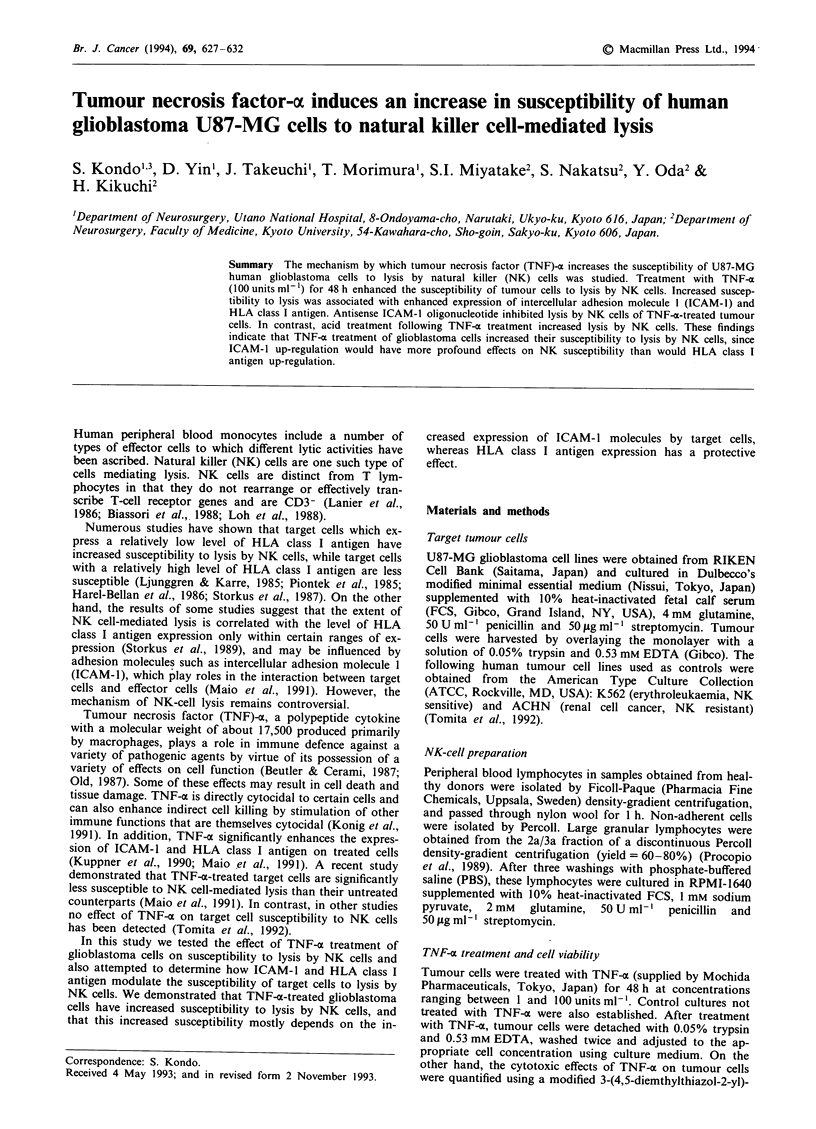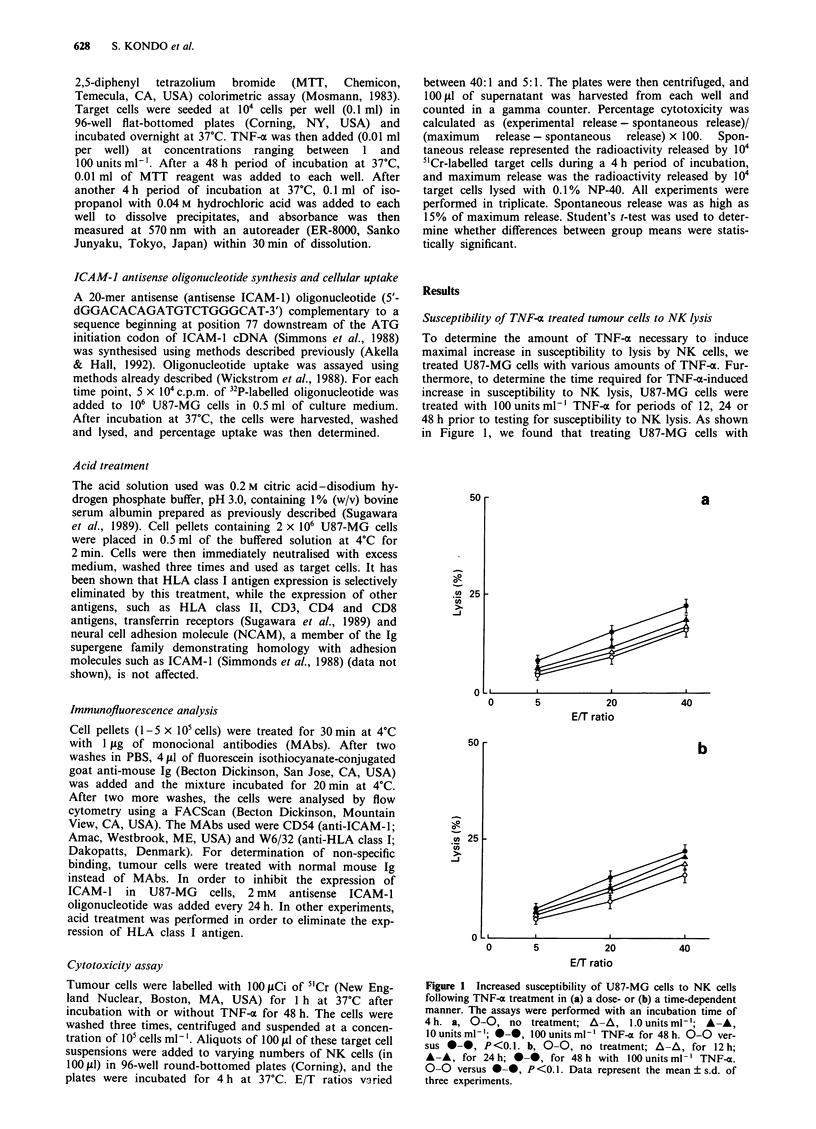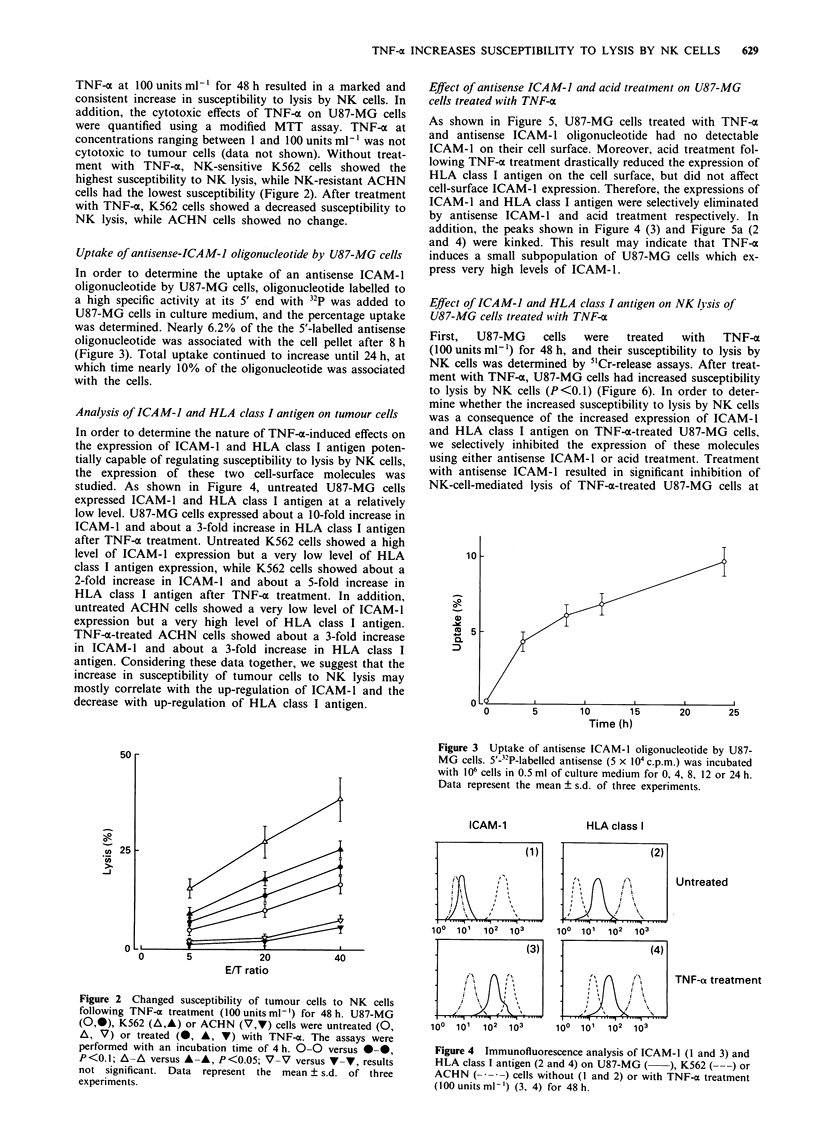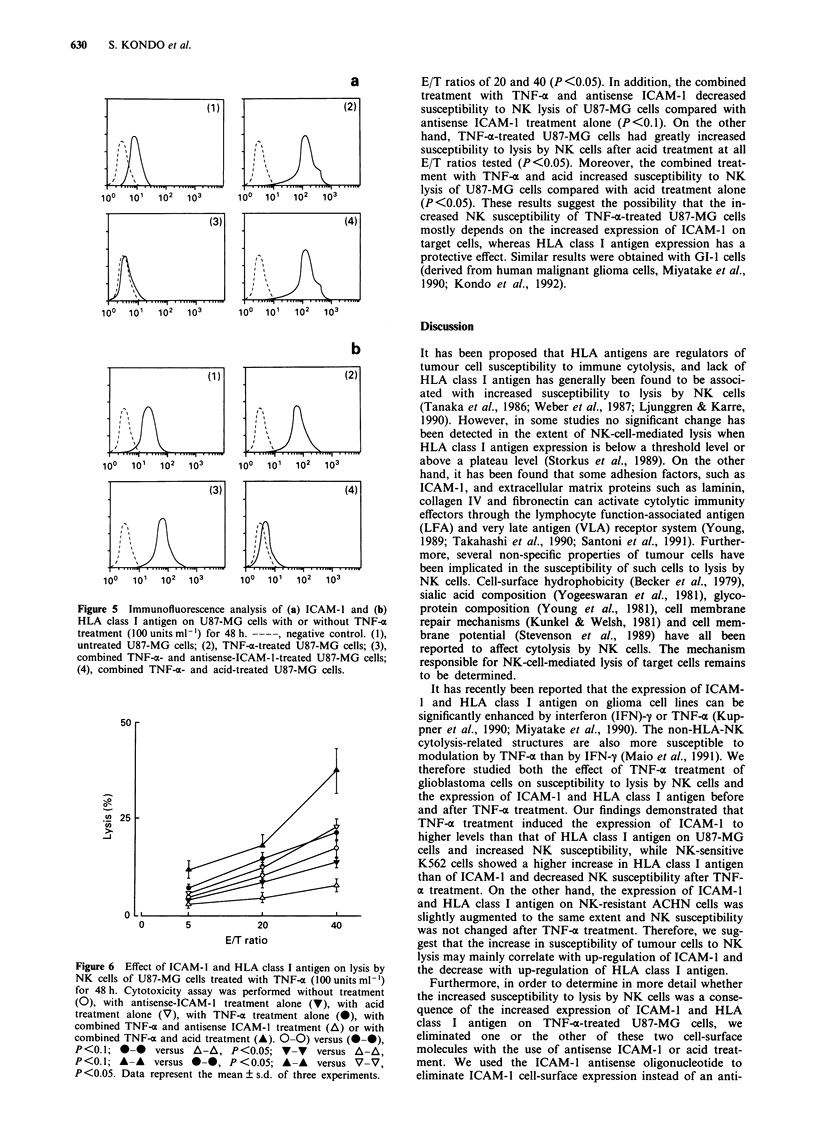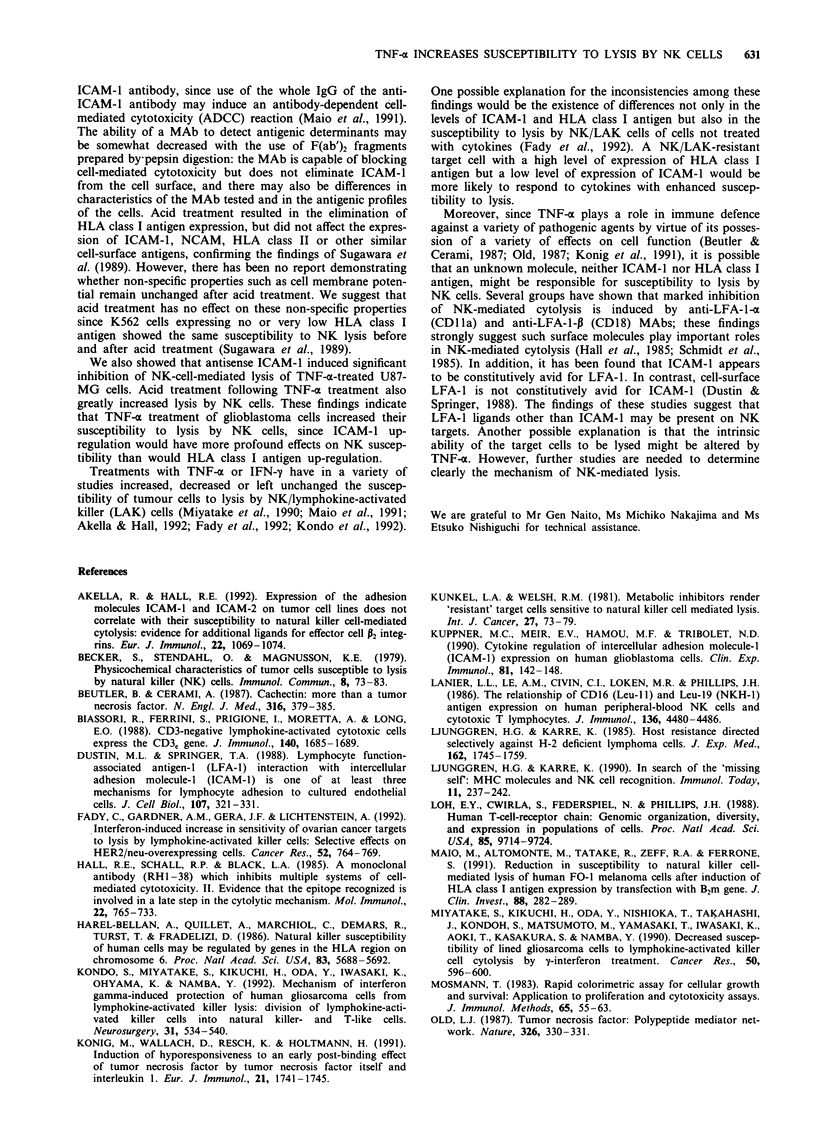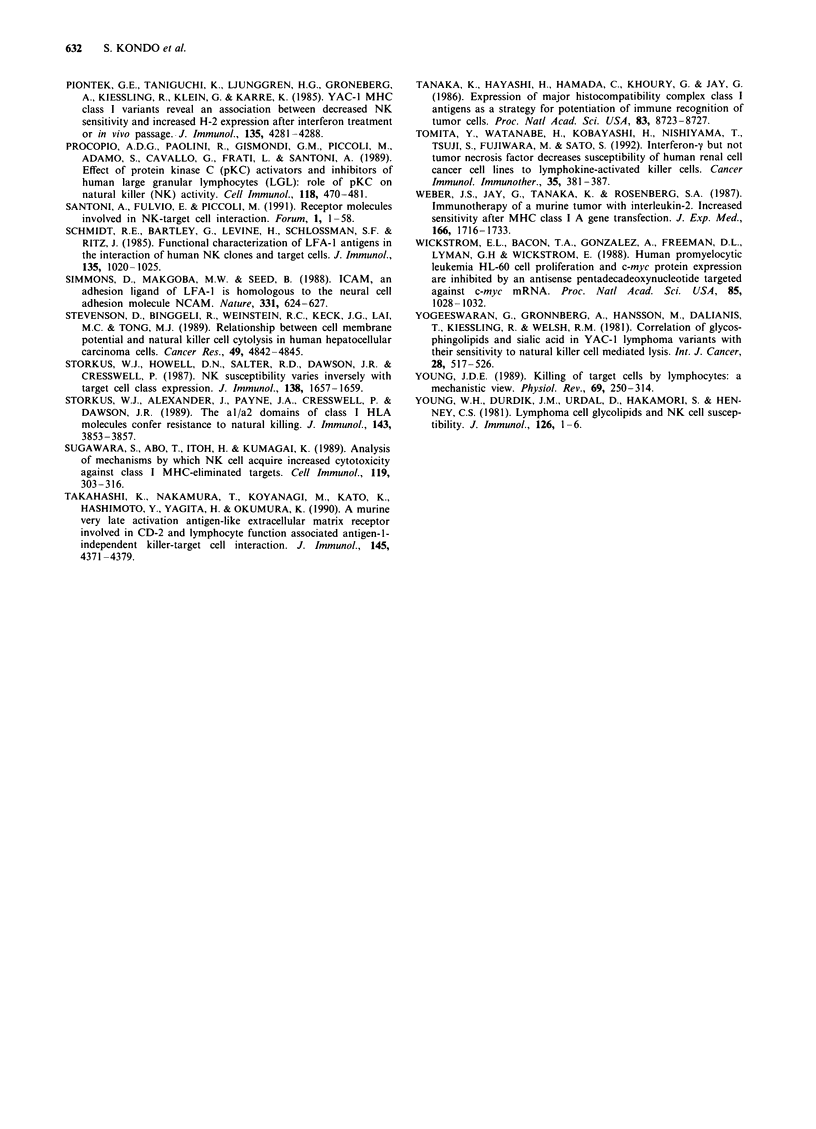# Tumour necrosis factor-alpha induces an increase in susceptibility of human glioblastoma U87-MG cells to natural killer cell-mediated lysis.

**DOI:** 10.1038/bjc.1994.123

**Published:** 1994-04

**Authors:** S. Kondo, D. Yin, J. Takeuchi, T. Morimura, S. I. Miyatake, S. Nakatsu, Y. Oda, H. Kikuchi

**Affiliations:** Department of Neurosurgery, Utano National Hospital, Kyoto, Japan.

## Abstract

The mechanism by which tumour necrosis factor (TNF)-alpha increases the susceptibility of U87-MG human glioblastoma cells to lysis by natural killer (NK) cells was studied. Treatment with TNF-alpha (100 units ml-1) for 48 h enhanced the susceptibility of tumour cells to lysis by NK cells. Increased susceptibility to lysis was associated with enhanced expression of intercellular adhesion molecule 1 (ICAM-1) and HLA class I antigen. Antisense ICAM-1 oligonucleotide inhibited lysis by NK cells of TNF-alpha-treated tumour cells. In contrast, acid treatment following TNF-alpha treatment increased lysis by NK cells. These findings indicate that TNF-alpha treatment of glioblastoma cells increased their susceptibility to lysis by NK cells, since ICAM-1 up-regulation would have more profound effects on NK susceptibility than would HLA class I antigen up-regulation.


Br. J. Cancer (1994), 69, 627–632 © Macmillan Press Ltd., 1994

### Tumour necrosis factor-α induces an increase in susceptibility of human glioblastoma U87-MG cells to natural killer cell-mediated lysis

S. Kondo[1,3], D. Yin[1], J. Takeuchi[1], T. Morimura[1], S.I. Miyatake[2], S. Nakatsu[2], Y. Oda[2] & H. Kikuchi[2]

[1]*Department of Neurosurgery, Utano National Hospital, 8-Ondoyama-cho, Narutaki, Ukyo-ku, Kyoto 616, Japan;* [2]*Department of Neurosurgery, Faculty of Medicine, Kyoto University, 54-Kawahara-cho, Sho-goin, Sakyo-ku, Kyoto 606, Japan.*

**Summary** The mechanism by which tumour necrosis factor (TNF)-α increases the susceptibility of U87-MG human glioblastoma cells to lysis by natural killer (NK) cells was studied. Treatment with TNF-α (100 units $ml^{-1}$) for 48 h enhanced the susceptibility of tumour cells to lysis by NK cells. Increased susceptibility to lysis was associated with enhanced expression of intercellular adhesion molecule 1 (ICAM-1) and HLA class I antigen. Antisense ICAM-1 oligonucleotide inhibited lysis by NK cells of TNF-α-treated tumour cells. In contrast, acid treatment following TNF-α treatment increased lysis by NK cells. These findings indicate that TNF-α treatment of glioblastoma cells increased their susceptibility to lysis by NK cells, since ICAM-1 up-regulation would have more profound effects on NK susceptibility than would HLA class I antigen up-regulation.

Human peripheral blood monocytes include a number of types of effector cells to which different lytic activities have been ascribed. Natural killer (NK) cells are one such type of cells mediating lysis. NK cells are distinct from T lymphocytes in that they do not rearrange or effectively transcribe T-cell receptor genes and are $CD3^-$ (Lanier *et al.*, 1986; Biassori *et al.*, 1988; Loh *et al.*, 1988).

Numerous studies have shown that target cells which express a relatively low level of HLA class I antigen have increased susceptibility to lysis by NK cells, while target cells with a relatively high level of HLA class I antigen are less susceptible (Ljunggren & Karre, 1985; Piontek *et al.*, 1985; Harel-Bellan *et al.*, 1986; Storkus *et al.*, 1987). On the other hand, the results of some studies suggest that the extent of NK cell-mediated lysis is correlated with the level of HLA class I antigen expression only within certain ranges of expression (Storkus *et al.*, 1989), and may be influenced by adhesion molecules such as intercellular adhesion molecule 1 (ICAM-1), which play roles in the interaction between target cells and effector cells (Maio *et al.*, 1991). However, the mechanism of NK-cell lysis remains controversial.

Tumour necrosis factor (TNF)-α, a polypeptide cytokine with a molecular weight of about 17,500 produced primarily by macrophages, plays a role in immune defence against a variety of pathogenic agents by virtue of its possession of a variety of effects on cell function (Beutler & Cerami, 1987; Old, 1987). Some of these effects may result in cell death and tissue damage. TNF-α is directly cytocidal to certain cells and can also enhance indirect cell killing by stimulation of other immune functions that are themselves cytocidal (Konig *et al.*, 1991). In addition, TNF-α significantly enhances the expression of ICAM-1 and HLA class I antigen on treated cells (Kuppner *et al.*, 1990; Maio *et al.*, 1991). A recent study demonstrated that TNF-α-treated target cells are significantly less susceptible to NK cell-mediated lysis than their untreated counterparts (Maio *et al.*, 1991). In contrast, in other studies no effect of TNF-α on target cell susceptibility to NK cells has been detected (Tomita *et al.*, 1992).

In this study we tested the effect of TNF-α treatment of glioblastoma cells on susceptibility to lysis by NK cells and also attempted to determine how ICAM-1 and HLA class I antigen modulate the susceptibility of target cells to lysis by NK cells. We demonstrated that TNF-α-treated glioblastoma cells have increased susceptibility to lysis by NK cells, and that this increased susceptibility mostly depends on the increased expression of ICAM-1 molecules by target cells, whereas HLA class I antigen expression has a protective effect.

Correspondence: S. Kondo.
Received 4 May 1993; and in revised form 2 November 1993.

### Materials and methods

### *Target tumour cells*

U87-MG glioblastoma cell lines were obtained from RIKEN Cell Bank (Saitama, Japan) and cultured in Dulbecco's modified minimal essential medium (Nissui, Tokyo, Japan) supplemented with 10% heat-inactivated fetal calf serum (FCS, Gibco, Grand Island, NY, USA), 4 mM glutamine, 50 U $ml^{-1}$ penicillin and 50 $\mu g\ ml^{-1}$ streptomycin. Tumour cells were harvested by overlaying the monolayer with a solution of 0.05% trypsin and 0.53 mM EDTA (Gibco). The following human tumour cell lines used as controls were obtained from the American Type Culture Collection (ATCC, Rockville, MD, USA): K562 (erythroleukaemia, NK sensitive) and ACHN (renal cell cancer, NK resistant) (Tomita *et al.*, 1992).

### *NK-cell preparation*

Peripheral blood lymphocytes in samples obtained from healthy donors were isolated by Ficoll-Paque (Pharmacia Fine Chemicals, Uppsala, Sweden) density-gradient centrifugation, and passed through nylon wool for 1 h. Non-adherent cells were isolated by Percoll. Large granular lymphocytes were obtained from the 2a/3a fraction of a discontinuous Percoll density-gradient centrifugation (yield = 60–80%) (Procopio *et al.*, 1989). After three washings with phosphate-buffered saline (PBS), these lymphocytes were cultured in RPMI-1640 supplemented with 10% heat-inactivated FCS, 1 mM sodium pyruvate, 2 mM glutamine, 50 U $ml^{-1}$ penicillin and 50 $\mu g\ ml^{-1}$ streptomycin.

### *TNF-α treatment and cell viability*

Tumour cells were treated with TNF-α (supplied by Mochida Pharmaceuticals, Tokyo, Japan) for 48 h at concentrations ranging between 1 and 100 units $ml^{-1}$. Control cultures not treated with TNF-α were also established. After treatment with TNF-α, tumour cells were detached with 0.05% trypsin and 0.53 mM EDTA, washed twice and adjusted to the appropriate cell concentration using culture medium. On the other hand, the cytotoxic effects of TNF-α on tumour cells were quantified using a modified 3-(4,5-diemthylthiazol-2-yl)-

2,5-diphenyl tetrazolium bromide (MTT, Chemicon, Temecula, CA, USA) colorimetric assay (Mosmann, 1983). Target cells were seeded at $10^4$ cells per well (0.1 ml) in 96-well flat-bottomed plates (Corning, NY, USA) and incubated overnight at 37°C. TNF-α was then added (0.01 ml per well) at concentrations ranging between 1 and 100 units $ml^{-1}$. After a 48 h period of incubation at 37°C, 0.01 ml of MTT reagent was added to each well. After another 4 h period of incubation at 37°C, 0.1 ml of isopropanol with 0.04 M hydrochloric acid was added to each well to dissolve precipitates, and absorbance was then measured at 570 nm with an autoreader (ER-8000, Sanko Junyaku, Tokyo, Japan) within 30 min of dissolution.

*ICAM-1 antisense oligonucleotide synthesis and cellular uptake*

A 20-mer antisense (antisense ICAM-1) oligonucleotide (5′-dGGACACAGATGTCTGGGCAT-3′) complementary to a sequence beginning at position 77 downstream of the ATG initiation codon of ICAM-1 cDNA (Simmons *et al.*, 1988) was synthesised using methods described previously (Akella & Hall, 1992). Oligonucleotide uptake was assayed using methods already described (Wickstrom *et al.*, 1988). For each time point, $5 \times 10^4$ c.p.m. of $^{32}$P-labelled oligonucleotide was added to $10^6$ U87-MG cells in 0.5 ml of culture medium. After incubation at 37°C, the cells were harvested, washed and lysed, and percentage uptake was then determined.

*Acid treatment*

The acid solution used was 0.2 M citric acid–disodium hydrogen phosphate buffer, pH 3.0, containing 1% (w/v) bovine serum albumin prepared as previously described (Sugawara *et al.*, 1989). Cell pellets containing $2 \times 10^6$ U87-MG cells were placed in 0.5 ml of the buffered solution at 4°C for 2 min. Cells were then immediately neutralised with excess medium, washed three times and used as target cells. It has been shown that HLA class I antigen expression is selectively eliminated by this treatment, while the expression of other antigens, such as HLA class II, CD3, CD4 and CD8 antigens, transferrin receptors (Sugawara *et al.*, 1989) and neural cell adhesion molecule (NCAM), a member of the Ig supergene family demonstrating homology with adhesion molecules such as ICAM-1 (Simmonds *et al.*, 1988) (data not shown), is not affected.

*Immunofluorescence analysis*

Cell pellets ($1-5 \times 10^5$ cells) were treated for 30 min at 4°C with 1 μg of monoclonal antibodies (MAbs). After two washes in PBS, 4 μl of fluorescein isothiocyanate-conjugated goat anti-mouse Ig (Becton Dickinson, San Jose, CA, USA) was added and the mixture incubated for 20 min at 4°C. After two more washes, the cells were analysed by flow cytometry using a FACScan (Becton Dickinson, Mountain View, CA, USA). The MAbs used were CD54 (anti-ICAM-1; Amac, Westbrook, ME, USA) and W6/32 (anti-HLA class I; Dakopatts, Denmark). For determination of non-specific binding, tumour cells were treated with normal mouse Ig instead of MAbs. In order to inhibit the expression of ICAM-1 in U87-MG cells, 2 mM antisense ICAM-1 oligonucleotide was added every 24 h. In other experiments, acid treatment was performed in order to eliminate the expression of HLA class I antigen.

*Cytotoxicity assay*

Tumour cells were labelled with 100 μCi of $^{51}$Cr (New England Nuclear, Boston, MA, USA) for 1 h at 37°C after incubation with or without TNF-α for 48 h. The cells were washed three times, centrifuged and suspended at a concentration of $10^5$ cells $ml^{-1}$. Aliquots of 100 μl of these target cell suspensions were added to varying numbers of NK cells (in 100 μl) in 96-well round-bottomed plates (Corning), and the plates were incubated for 4 h at 37°C. E/T ratios varied between 40:1 and 5:1. The plates were then centrifuged, and 100 μl of supernatant was harvested from each well and counted in a gamma counter. Percentage cytotoxicity was calculated as (experimental release – spontaneous release)/(maximum release – spontaneous release) × 100. Spontaneous release represented the radioactivity released by $10^4$ $^{51}$Cr-labelled target cells during a 4 h period of incubation, and maximum release was the radioactivity released by $10^4$ target cells lysed with 0.1% NP-40. All experiments were performed in triplicate. Spontaneous release was as high as 15% of maximum release. Student's *t*-test was used to determine whether differences between group means were statistically significant.

### Results

*Susceptibility of TNF-α treated tumour cells to NK lysis*

To determine the amount of TNF-α necessary to induce maximal increase in susceptibility to lysis by NK cells, we treated U87-MG cells with various amounts of TNF-α. Furthermore, to determine the time required for TNF-α-induced increase in susceptibility to NK lysis, U87-MG cells were treated with 100 units $ml^{-1}$ TNF-α for periods of 12, 24 or 48 h prior to testing for susceptibility to NK lysis. As shown in Figure 1, we found that treating U87-MG cells with

**Figure 1** Increased susceptibility of U87-MG cells to NK cells following TNF-α treatment in (**a**) a dose- or (**b**) a time-dependent manner. The assays were performed with an incubation time of 4 h. **a**, ○–○, no treatment; △–△, 1.0 units $ml^{-1}$; ▲–▲, 10 units $ml^{-1}$; ●–●, 100 units $ml^{-1}$ TNF-α for 48 h. ○–○ versus ●–●, $P<0.1$. **b**, ○–○, no treatment; △–△, for 12 h; ▲–▲, for 24 h; ●–●, for 48 h with 100 units $ml^{-1}$ TNF-α. ○–○ versus ●–●, $P<0.1$. Data represent the mean ± s.d. of three experiments.

TNF-α at 100 units ml$^{-1}$ for 48 h resulted in a marked and consistent increase in susceptibility to lysis by NK cells. In addition, the cytotoxic effects of TNF-α on U87-MG cells were quantified using a modified MTT assay. TNF-α at concentrations ranging between 1 and 100 units ml$^{-1}$ was not cytotoxic to tumour cells (data not shown). Without treatment with TNF-α, NK-sensitive K562 cells showed the highest susceptibility to NK lysis, while NK-resistant ACHN cells had the lowest susceptibility (Figure 2). After treatment with TNF-α, K562 cells showed a decreased susceptibility to NK lysis, while ACHN cells showed no change.

*Uptake of antisense-ICAM-1 oligonucleotide by U87-MG cells*

In order to determine the uptake of an antisense ICAM-1 oligonucleotide by U87-MG cells, oligonucleotide labelled to a high specific activity at its 5′ end with $^{32}P$ was added to U87-MG cells in culture medium, and the percentage uptake was determined. Nearly 6.2% of the the 5′-labelled antisense oligonucleotide was associated with the cell pellet after 8 h (Figure 3). Total uptake continued to increase until 24 h, at which time nearly 10% of the oligonucleotide was associated with the cells.

*Analysis of ICAM-1 and HLA class I antigen on tumour cells*

In order to determine the nature of TNF-α-induced effects on the expression of ICAM-1 and HLA class I antigen potentially capable of regulating susceptibility to lysis by NK cells, the expression of these two cell-surface molecules was studied. As shown in Figure 4, untreated U87-MG cells expressed ICAM-1 and HLA class I antigen at a relatively low level. U87-MG cells expressed about a 10-fold increase in ICAM-1 and about a 3-fold increase in HLA class I antigen after TNF-α treatment. Untreated K562 cells showed a high level of ICAM-1 expression but a very low level of HLA class I antigen expression, while K562 cells showed about a 2-fold increase in ICAM-1 and about a 5-fold increase in HLA class I antigen after TNF-α treatment. In addition, untreated ACHN cells showed a very low level of ICAM-1 expression but a very high level of HLA class I antigen. TNF-α-treated ACHN cells showed about a 3-fold increase in ICAM-1 and about a 3-fold increase in HLA class I antigen. Considering these data together, we suggest that the increase in susceptibility of tumour cells to NK lysis may mostly correlate with the up-regulation of ICAM-1 and the decrease with up-regulation of HLA class I antigen.

**Figure 2** Changed susceptibility of tumour cells to NK cells following TNF-α treatment (100 units ml$^{-1}$) for 48 h. U87-MG (O,●), K562 (Δ,▲) or ACHN (∇,▼) cells were untreated (O, Δ, ∇) or treated (●, ▲, ▼) with TNF-α. The assays were performed with an incubation time of 4 h. O-O versus ●-●, $P<0.1$; Δ-Δ versus ▲-▲, $P<0.05$; ∇-∇ versus ▼-▼, results not significant. Data represent the mean ± s.d. of three experiments.

*Effect of antisense ICAM-1 and acid treatment on U87-MG cells treated with TNF-α*

As shown in Figure 5, U87-MG cells treated with TNF-α and antisense ICAM-1 oligonucleotide had no detectable ICAM-1 on their cell surface. Moreover, acid treatment following TNF-α treatment drastically reduced the expression of HLA class I antigen on the cell surface, but did not affect cell-surface ICAM-1 expression. Therefore, the expressions of ICAM-1 and HLA class I antigen were selectively eliminated by antisense ICAM-1 and acid treatment respectively. In addition, the peaks shown in Figure 4 (3) and Figure 5a (2 and 4) were kinked. This result may indicate that TNF-α induces a small subpopulation of U87-MG cells which express very high levels of ICAM-1.

*Effect of ICAM-1 and HLA class I antigen on NK lysis of U87-MG cells treated with TNF-α*

First, U87-MG cells were treated with TNF-α (100 units ml$^{-1}$) for 48 h, and their susceptibility to lysis by NK cells was determined by $^{51}Cr$-release assays. After treatment with TNF-α, U87-MG cells had increased susceptibility to lysis by NK cells ($P<0.1$) (Figure 6). In order to determine whether the increased susceptibility to lysis by NK cells was a consequence of the increased expression of ICAM-1 and HLA class I antigen on TNF-α-treated U87-MG cells, we selectively inhibited the expression of these molecules using either antisense ICAM-1 or acid treatment. Treatment with antisense ICAM-1 resulted in significant inhibition of NK-cell-mediated lysis of TNF-α-treated U87-MG cells at

**Figure 3** Uptake of antisense ICAM-1 oligonucleotide by U87-MG cells. 5′-$^{32}P$-labelled antisense ($5 \times 10^4$ c.p.m.) was incubated with $10^6$ cells in 0.5 ml of culture medium for 0, 4, 8, 12 or 24 h. Data represent the mean ± s.d. of three experiments.

**Figure 4** Immunofluorescence analysis of ICAM-1 (1 and 3) and HLA class I antigen (2 and 4) on U87-MG (——), K562 (– – –) or ACHN (-·-·-) cells without (1 and 2) or with TNF-α treatment (100 units ml$^{-1}$) (3, 4) for 48 h.

**Figure 5** Immunofluorescence analysis of (**a**) ICAM-1 and (**b**) HLA class I antigen on U87-MG cells with or without TNF-α treatment (100 units $ml^{-1}$) for 48 h. ----, negative control. (1), untreated U87-MG cells; (2), TNF-α-treated U87-MG cells; (3), combined TNF-α- and antisense-ICAM-1-treated U87-MG cells; (4), combined TNF-α- and acid-treated U87-MG cells.

**Figure 6** Effect of ICAM-1 and HLA class I antigen on lysis by NK cells of U87-MG cells treated with TNF-α (100 units $ml^{-1}$) for 48 h. Cytotoxicity assay was performed without treatment (○), with antisense-ICAM-1 treatment alone (▼), with acid treatment alone (▽), with TNF-α treatment alone (●), with combined TNF-α and antisense ICAM-1 treatment (△) or with combined TNF-α and acid treatment (▲). ○-○) versus (●-●), $P<0.1$; ●-● versus △-△, $P<0.05$; ▼-▼ versus △-△, $P<0.1$; ▲-▲ versus ●-●, $P<0.05$; ▲-▲ versus ▽-▽, $P<0.05$. Data represent the mean ± s.d. of three experiments.

E/T ratios of 20 and 40 ($P<0.05$). In addition, the combined treatment with TNF-α and antisense ICAM-1 decreased susceptibility to NK lysis of U87-MG cells compared with antisense ICAM-1 treatment alone ($P<0.1$). On the other hand, TNF-α-treated U87-MG cells had greatly increased susceptibility to lysis by NK cells after acid treatment at all E/T ratios tested ($P<0.05$). Moreover, the combined treatment with TNF-α and acid increased susceptibility to NK lysis of U87-MG cells compared with acid treatment alone ($P<0.05$). These results suggest the possibility that the increased NK susceptibility of TNF-α-treated U87-MG cells mostly depends on the increased expression of ICAM-1 on target cells, whereas HLA class I antigen expression has a protective effect. Similar results were obtained with GI-1 cells (derived from human malignant glioma cells, Miyatake et al., 1990; Kondo et al., 1992).

### Discussion

It has been proposed that HLA antigens are regulators of tumour cell susceptibility to immune cytolysis, and lack of HLA class I antigen has generally been found to be associated with increased susceptibility to lysis by NK cells (Tanaka et al., 1986; Weber et al., 1987; Ljunggren & Karre, 1990). However, in some studies no significant change has been detected in the extent of NK-cell-mediated lysis when HLA class I antigen expression is below a threshold level or above a plateau level (Storkus et al., 1989). On the other hand, it has been found that some adhesion factors, such as ICAM-1, and extracellular matrix proteins such as laminin, collagen IV and fibronectin can activate cytolytic immunity effectors through the lymphocyte function-associated antigen (LFA) and very late antigen (VLA) receptor system (Young, 1989; Takahashi et al., 1990; Santoni et al., 1991). Furthermore, several non-specific properties of tumour cells have been implicated in the susceptibility of such cells to lysis by NK cells. Cell-surface hydrophobicity (Becker et al., 1979), sialic acid composition (Yogeeswaran et al., 1981), glycoprotein composition (Young et al., 1981), cell membrane repair mechanisms (Kunkel & Welsh, 1981) and cell membrane potential (Stevenson et al., 1989) have all been reported to affect cytolysis by NK cells. The mechanism responsible for NK-cell-mediated lysis of target cells remains to be determined.

It has recently been reported that the expression of ICAM-1 and HLA class I antigen on glioma cell lines can be significantly enhanced by interferon (IFN)-γ or TNF-α (Kuppner et al., 1990; Miyatake et al., 1990). The non-HLA-NK cytolysis-related structures are also more susceptible to modulation by TNF-α than by IFN-γ (Maio et al., 1991). We therefore studied both the effect of TNF-α treatment of glioblastoma cells on susceptibility to lysis by NK cells and the expression of ICAM-1 and HLA class I antigen before and after TNF-α treatment. Our findings demonstrated that TNF-α treatment induced the expression of ICAM-1 to higher levels than that of HLA class I antigen on U87-MG cells and increased NK susceptibility, while NK-sensitive K562 cells showed a higher increase in HLA class I antigen than of ICAM-1 and decreased NK susceptibility after TNF-α treatment. On the other hand, the expression of ICAM-1 and HLA class I antigen on NK-resistant ACHN cells was slightly augmented to the same extent and NK susceptibility was not changed after TNF-α treatment. Therefore, we suggest that the increase in susceptibility of tumour cells to NK lysis may mainly correlate with up-regulation of ICAM-1 and the decrease with up-regulation of HLA class I antigen.

Furthermore, in order to determine in more detail whether the increased susceptibility to lysis by NK cells was a consequence of the increased expression of ICAM-1 and HLA class I antigen on TNF-α-treated U87-MG cells, we eliminated one or the other of these two cell-surface molecules with the use of antisense ICAM-1 or acid treatment. We used the ICAM-1 antisense oligonucleotide to eliminate ICAM-1 cell-surface expression instead of an anti-

ICAM-1 antibody, since use of the whole IgG of the anti-ICAM-1 antibody may induce an antibody-dependent cell-mediated cytotoxicity (ADCC) reaction (Maio *et al.*, 1991). The ability of a MAb to detect antigenic determinants may be somewhat decreased with the use of $F(ab')_2$ fragments prepared by pepsin digestion: the MAb is capable of blocking cell-mediated cytotoxicity but does not eliminate ICAM-1 from the cell surface, and there may also be differences in characteristics of the MAb tested and in the antigenic profiles of the cells. Acid treatment resulted in the elimination of HLA class I antigen expression, but did not affect the expression of ICAM-1, NCAM, HLA class II or other similar cell-surface antigens, confirming the findings of Sugawara *et al.* (1989). However, there has been no report demonstrating whether non-specific properties such as cell membrane potential remain unchanged after acid treatment. We suggest that acid treatment has no effect on these non-specific properties since K562 cells expressing no or very low HLA class I antigen showed the same susceptibility to NK lysis before and after acid treatment (Sugawara *et al.*, 1989).

We also showed that antisense ICAM-1 induced significant inhibition of NK-cell-mediated lysis of TNF-α-treated U87-MG cells. Acid treatment following TNF-α treatment also greatly increased lysis by NK cells. These findings indicate that TNF-α treatment of glioblastoma cells increased their susceptibility to lysis by NK cells, since ICAM-1 up-regulation would have more profound effects on NK susceptibility than would HLA class I antigen up-regulation.

Treatments with TNF-α or IFN-γ have in a variety of studies increased, decreased or left unchanged the susceptibility of tumour cells to lysis by NK/lymphokine-activated killer (LAK) cells (Miyatake *et al.*, 1990; Maio *et al.*, 1991; Akella & Hall, 1992; Fady *et al.*, 1992; Kondo *et al.*, 1992). One possible explanation for the inconsistencies among these findings would be the existence of differences not only in the levels of ICAM-1 and HLA class I antigen but also in the susceptibility to lysis by NK/LAK cells of cells not treated with cytokines (Fady *et al.*, 1992). A NK/LAK-resistant target cell with a high level of expression of HLA class I antigen but a low level of expression of ICAM-1 would be more likely to respond to cytokines with enhanced susceptibility to lysis.

Moreover, since TNF-α plays a role in immune defence against a variety of pathogenic agents by virtue of its possession of a variety of effects on cell function (Beutler & Cerami, 1987; Old, 1987; Konig *et al.*, 1991), it is possible that an unknown molecule, neither ICAM-1 nor HLA class I antigen, might be responsible for susceptibility to lysis by NK cells. Several groups have shown that marked inhibition of NK-mediated cytolysis is induced by anti-LFA-1-α (CD11a) and anti-LFA-1-β (CD18) MAbs; these findings strongly suggest such surface molecules play important roles in NK-mediated cytolysis (Hall *et al.*, 1985; Schmidt *et al.*, 1985). In addition, it has been found that ICAM-1 appears to be constitutively avid for LFA-1. In contrast, cell-surface LFA-1 is not constitutively avid for ICAM-1 (Dustin & Springer, 1988). The findings of these studies suggest that LFA-1 ligands other than ICAM-1 may be present on NK targets. Another possible explanation is that the intrinsic ability of the target cells to be lysed might be altered by TNF-α. However, further studies are needed to determine clearly the mechanism of NK-mediated lysis.

We are grateful to Mr Gen Naito, Ms Michiko Nakajima and Ms Etsuko Nishiguchi for technical assistance.

### References

AKELLA, R. & HALL, R.E. (1992). Expression of the adhesion molecules ICAM-1 and ICAM-2 on tumor cell lines does not correlate with their susceptibility to natural killer cell-mediated cytolysis: evidence for additional ligands for effector cell $\beta_2$ integrins. *Eur. J. Immunol.*, **22**, 1069–1074.

BECKER, S., STENDAHL, O. & MAGNUSSON, K.E. (1979). Physicochemical characteristics of tumor cells susceptible to lysis by natural killer (NK) cells. *Immunol. Commun.*, **8**, 73–83.

BEUTLER, B. & CERAMI, A. (1987). Cachectin: more than a tumor necrosis factor. *N. Engl. J. Med.*, **316**, 379–385.

BIASSORI, R., FERRINI, S., PRIGIONE, I., MORETTA, A. & LONG, E.O. (1988). CD3-negative lymphokine-activated cytotoxic cells express the $CD3_\epsilon$ gene. *J. Immunol.*, **140**, 1685–1689.

DUSTIN, M.L. & SPRINGER, T.A. (1988). Lymphocyte function-associated antigen-1 (LFA-1) interaction with intercellular adhesion molecule-1 (ICAM-1) is one of at least three mechanisms for lymphocyte adhesion to cultured endothelial cells. *J. Cell Biol.*, **107**, 321–331.

FADY, C., GARDNER, A.M., GERA, J.F. & LICHTENSTEIN, A. (1992). Interferon-induced increase in sensitivity of ovarian cancer targets to lysis by lymphokine-activated killer cells: Selective effects on HER2/neu-overexpressing cells. *Cancer Res.*, **52**, 764–769.

HALL, R.E., SCHALL, R.P. & BLACK, L.A. (1985). A monoclonal antibody (RH1–38) which inhibits multiple systems of cell-mediated cytotoxicity. II. Evidence that the epitope recognized is involved in a late step in the cytolytic mechanism. *Mol. Immunol.*, **22**, 765–733.

HAREL-BELLAN, A., QUILLET, A., MARCHIOL, C., DEMARS, R., TURST, T. & FRADELIZI, D. (1986). Natural killer susceptibility of human cells may be regulated by genes in the HLA region on chromosome 6. *Proc. Natl Acad. Sci. USA*, **83**, 5688–5692.

KONDO, S., MIYATAKE, S., KIKUCHI, H., ODA, Y., IWASAKI, K., OHYAMA, K. & NAMBA, Y. (1992). Mechanism of interferon gamma-induced protection of human gliosarcoma cells from lymphokine-activated killer lysis: division of lymphokine-activated killer cells into natural killer- and T-like cells. *Neurosurgery*, **31**, 534–540.

KONIG, M., WALLACH, D., RESCH, K. & HOLTMANN, H. (1991). Induction of hyporesponsiveness to an early post-binding effect of tumor necrosis factor by tumor necrosis factor itself and interleukin 1. *Eur. J. Immunol.*, **21**, 1741–1745.

KUNKEL, L.A. & WELSH, R.M. (1981). Metabolic inhibitors render 'resistant' target cells sensitive to natural killer cell mediated lysis. *Int. J. Cancer*, **27**, 73–79.

KUPPNER, M.C., MEIR, E.V., HAMOU, M.F. & TRIBOLET, N.D. (1990). Cytokine regulation of intercellular adhesion molecule-1 (ICAM-1) expression on human glioblastoma cells. *Clin. Exp. Immunol.*, **81**, 142–148.

LANIER, L.L., LE, A.M., CIVIN, C.I., LOKEN, M.R. & PHILLIPS, J.H. (1986). The relationship of CD16 (Leu-11) and Leu-19 (NKH-1) antigen expression on human peripheral-blood NK cells and cytotoxic T lymphocytes. *J. Immunol.*, **136**, 4480–4486.

LJUNGGREN, H.G. & KARRE, K. (1985). Host resistance directed selectively against H-2 deficient lymphoma cells. *J. Exp. Med.*, **162**, 1745–1759.

LJUNGGREN, H.G. & KARRE, K. (1990). In search of the 'missing self': MHC molecules and NK cell recognition. *Immunol. Today*, **11**, 237–242.

LOH, E.Y., CWIRLA, S., FEDERSPIEL, N. & PHILLIPS, J.H. (1988). Human T-cell-receptor chain: Genomic organization, diversity, and expression in populations of cells. *Proc. Natl Acad. Sci. USA*, **85**, 9714–9724.

MAIO, M., ALTOMONTE, M., TATAKE, R., ZEFF, R.A. & FERRONE, S. (1991). Reduction in susceptibility to natural killer cell-mediated lysis of human FO-1 melanoma cells after induction of HLA class I antigen expression by transfection with $B_2m$ gene. *J. Clin. Invest.*, **88**, 282–289.

MIYATAKE, S., KIKUCHI, H., ODA, Y., NISHIOKA, T., TAKAHASHI, J., KONDOH, S., MATSUMOTO, M., YAMASAKI, T., IWASAKI, K., AOKI, T., KASAKURA, S. & NAMBA, Y. (1990). Decreased susceptibility of lined gliosarcoma cells to lymphokine-activated killer cell cytolysis by γ-interferon treatment. *Cancer Res.*, **50**, 596–600.

MOSMANN, T. (1983). Rapid colorimetric assay for cellular growth and survival: Application to proliferation and cytotoxicity assays. *J. Immunol. Methods*, **65**, 55–63.

OLD, L.J. (1987). Tumor necrosis factor: Polypeptide mediator network. *Nature*, **326**, 330–331.

PIONTEK, G.E., TANIGUCHI, K., LJUNGGREN, H.G., GRONEBERG, A., KIESSLING, R., KLEIN, G. & KARRE, K. (1985). YAC-1 MHC class I variants reveal an association between decreased NK sensitivity and increased H-2 expression after interferon treatment or *in vivo* passage. *J. Immunol.*, **135**, 4281–4288.

PROCOPIO, A.D.G., PAOLINI, R., GISMONDI, G.M., PICCOLI, M., ADAMO, S., CAVALLO, G., FRATI, L. & SANTONI, A. (1989). Effect of protein kinase C (pKC) activators and inhibitors of human large granular lymphocytes (LGL): role of pKC on natural killer (NK) activity. *Cell Immunol.*, **118**, 470–481.

SANTONI, A., FULVIO, E. & PICCOLI, M. (1991). Receptor molecules involved in NK-target cell interaction. *Forum*, **1**, 1–58.

SCHMIDT, R.E., BARTLEY, G., LEVINE, H., SCHLOSSMAN, S.F. & RITZ, J. (1985). Functional characterization of LFA-1 antigens in the interaction of human NK clones and target cells. *J. Immunol.*, **135**, 1020–1025.

SIMMONS, D., MAKGOBA, M.W. & SEED, B. (1988). ICAM, an adhesion ligand of LFA-1 is homologous to the neural cell adhesion molecule NCAM. *Nature*, **331**, 624-627.

STEVENSON, D., BINGGELI, R., WEINSTEIN, R.C., KECK, J.G., LAI, M.C. & TONG, M.J. (1989). Relationship between cell membrane potential and natural killer cell cytolysis in human hepatocellular carcinoma cells. *Cancer Res.*, **49**, 4842–4845.

STORKUS, W.J., HOWELL, D.N., SALTER, R.D., DAWSON, J.R. & CRESSWELL, P. (1987). NK susceptibility varies inversely with target cell class expression. *J. Immunol.*, **138**, 1657–1659.

STORKUS, W.J., ALEXANDER, J., PAYNE, J.A., CRESSWELL, P. & DAWSON, J.R. (1989). The a1/a2 domains of class I HLA molecules confer resistance to natural killing. *J. Immunol.*, **143**, 3853–3857.

SUGAWARA, S., ABO, T., ITOH, H. & KUMAGAI, K. (1989). Analysis of mechanisms by which NK cell acquire increased cytotoxicity against class I MHC-eliminated targets. *Cell Immunol.*, **119**, 303–316.

TAKAHASHI, K., NAKAMURA, T., KOYANAGI, M., KATO, K., HASHIMOTO, Y., YAGITA, H. & OKUMURA, K. (1990). A murine very late activation antigen-like extracellular matrix receptor involved in CD-2 and lymphocyte function associated antigen-1-independent killer-target cell interaction. *J. Immunol.*, **145**, 4371–4379.

TANAKA, K., HAYASHI, H., HAMADA, C., KHOURY, G. & JAY, G. (1986). Expression of major histocompatibility complex class I antigens as a strategy for potentiation of immune recognition of tumor cells. *Proc. Natl Acad. Sci. USA*, **83**, 8723–8727.

TOMITA, Y., WATANABE, H., KOBAYASHI, H., NISHIYAMA, T., TSUJI, S., FUJIWARA, M. & SATO, S. (1992). Interferon-γ but not tumor necrosis factor decreases susceptibility of human renal cell cancer cell lines to lymphokine-activated killer cells. *Cancer Immunol. Immunother.*, **35**, 381–387.

WEBER, J.S., JAY, G., TANAKA, K. & ROSENBERG, S.A. (1987). Immunotherapy of a murine tumor with interleukin-2. Increased sensitivity after MHC class I A gene transfection. *J. Exp. Med.*, **166**, 1716–1733.

WICKSTROM, E.L., BACON, T.A., GONZALEZ, A., FREEMAN, D.L., LYMAN, G.H & WICKSTROM, E. (1988). Human promyelocytic leukemia HL-60 cell proliferation and c-*myc* protein expression are inhibited by an antisense pentadecadeoxynucleotide targeted against c-*myc* mRNA. *Proc. Natl Acad. Sci. USA*, **85**, 1028–1032.

YOGEESWARAN, G., GRONNBERG, A., HANSSON, M., DALIANIS, T., KIESSLING, R. & WELSH, R.M. (1981). Correlation of glycosphingolipids and sialic acid in YAC-1 lymphoma variants with their sensitivity to natural killer cell mediated lysis. *Int. J. Cancer*, **28**, 517–526.

YOUNG, J.D.E. (1989). Killing of target cells by lymphocytes: a mechanistic view. *Physiol. Rev.*, **69**, 250–314.

YOUNG, W.H., DURDIK, J.M., URDAL, D., HAKAMORI, S. & HENNEY, C.S. (1981). Lymphoma cell glycolipids and NK cell susceptibility. *J. Immunol.*, **126**, 1–6.